# Comprehensive screening of genomic and metagenomic data reveals a large diversity of tetracycline resistance genes

**DOI:** 10.1099/mgen.0.000455

**Published:** 2020-10-30

**Authors:** Fanny Berglund, Maria-Elisabeth Böhm, Anton Martinsson, Stefan Ebmeyer, Tobias Österlund, Anna Johnning, D. G. Joakim Larsson, Erik Kristiansson

**Affiliations:** ^1^​ Department of Mathematical Sciences, Chalmers University of Technology and University of Gothenburg, Gothenburg, Sweden; ^2^​ Centre for Antibiotic Resistance Research (CARe), University of Gothenburg, Gothenburg, Sweden; ^3^​ Department of Infectious Diseases, Institute of Biomedicine, Sahlgrenska Academy, University of Gothenburg, Gothenburg, Sweden; ^4^​ Department of Systems and Data Analysis, Fraunhofer-Chalmers Centre, Chalmers Science Park, Gothenburg, Sweden

**Keywords:** antibiotic resistance, hidden Markov model, metagenomics, microbiome, resistome, tetracycline resistance

## Abstract

Tetracyclines are broad-spectrum antibiotics used to prevent or treat a variety of bacterial infections. Resistance is often mediated through mobile resistance genes, which encode one of the three main mechanisms: active efflux, ribosomal target protection or enzymatic degradation. In the last few decades, a large number of new tetracycline-resistance genes have been discovered in clinical settings. These genes are hypothesized to originate from environmental and commensal bacteria, but the diversity of tetracycline-resistance determinants that have not yet been mobilized into pathogens is unknown. In this study, we aimed to characterize the potential tetracycline resistome by screening genomic and metagenomic data for novel resistance genes. By using probabilistic models, we predicted 1254 unique putative tetracycline resistance genes, representing 195 gene families (<70 % amino acid sequence identity), whereof 164 families had not been described previously. Out of 17 predicted genes selected for experimental verification, 7 induced a resistance phenotype in an *Escherichia coli* host. Several of the predicted genes were located on mobile genetic elements or in regions that indicated mobility, suggesting that they easily can be shared between bacteria. Furthermore, phylogenetic analysis indicated several events of horizontal gene transfer between bacterial phyla. Our results also suggested that acquired efflux pumps originate from proteobacterial species, while ribosomal protection genes have been mobilized from *Firmicutes* and *Actinobacteria *. This study significantly expands the knowledge of known and putatively novel tetracycline resistance genes, their mobility and evolutionary history. The study also provides insights into the unknown resistome and genes that may be encountered in clinical settings in the future.

## Data Summary

The data analysed in this study consisted of pre-existing datasets, which are specified in Table 1 and throughout the text. The genes used to construct the hidden Markov models are listed in Table S1 (available with the online version of this article). The new genes predicted in this work are listed in Table S3, together with their respective protein sequences. The fARGene method is publicly available at https://github.com/fannyhb/fargene.

**Table 1.** Predicted tetracycline resistance genes

Dataset	Size (nt)	Efflux		Ribosomal protection		Enzymatic	
	Genes*	Families (new/total)†	Genes*	Families (new/total)†	Genes*	Families (new/total)†
Genomic							
NCBI RefSeq [77]	2.78×10^10^	137	16/29	232	51/57	20	9/11
NCBI plasmids [77]	1.07×10^9^	54	2/13	35	3/7	3	0/1
NCBI nt [19]	1.66×10^11^	289	30/45	563	77/87	76	14/20
NCBI environmental [19]	1.09×10^11^	23	7/15	317	17/23	17	3/6
HMP genomic [41]	6.83×10^9^	20	2/8	105	7/12	4	1/3
**Metagenomic**							
Human gut [47]	2.80×10^11^	3	0/2	13	0/5	2	0/2
HMP metagenome [45]	3.51×10^12^	3	0/3	10	3/5	2	0/2
Pig gut [44]	1.74×10^12^	9	0/7	13	6/10	1	0/1
WWTPs [42]	4.82×10^11^	11	4/8	14	5/12	3	2/3
Pune river [43]	3.93×10^11^	5	1/5	19	8/13	1	0/1
Polluted lake [50]	6.76×10^9^	0	0	0	0	0	0
Polluted river [46]	2.86×10^10^	3	0/2	2	1/2	1	0/1
Oil spill [49]	3.39×10^11^	0	0	0	0	0	0
Tara Ocean [48]	4.89×10^12^	4	2/3	0	0	4	2/3
Well water [51]	7.62×10^10^	0	0	0	0	0	0
Soil [51]	5.05×10^10^	0	0	0	0	0	0
**Total‡**	**1.21×10^13^**	**331**	**38/53**	**927**	**106/116**	**96**	**20/26**

*Non-redundant genes.

†Amino acid sequence identity cut-off of 70 %.

‡Non redundant genes/gene families.

**Impact Statement**

Tetracyclines are one of the most widely used classes of antibiotics and crucial for treating many forms of infection. However, the incidence of tetracycline resistant bacteria is increasing globally, which is to a large extent caused by new variants of tetracycline-resistance genes encountered in pathogens. These genes are hypothesized to originate from an unexplored reservoir of tetracycline resistance genes maintained by environmental bacteria. The size and content of this reservoir are unknown and, thus, we do not know the evolutionary history of the known tetracycline resistance genes and which novel genes we may encounter in the future. In this study, we have characterized a large number of new putative tetracycline resistance genes not yet encountered in clinical settings. Phylogenetic analysis of the predicted and previously known tetracycline resistance genes has given new insights about their evolutionary origin and mobility patterns. This study significantly expands the known tetracycline resistome, and describes which resistance genes may be mobilized and transferred into pathogens in the future.

## Introduction

Tetracyclines are broad-spectrum antibiotics widely used to treat a broad range of bacterial infections since their discovery and introduction as clinical agents in the 1940s. Efficiency against many forms of both Gram-negative and Gram-positive bacteria in combination with relatively few side effects have made tetracyclines one of the most extensively used classes of antibiotics, and the most used class for animals [1]. In addition to clinical use, tetracyclines are commonly used in subtherapeutic doses to induce growth-promotion in, for example, swine and poultry [2]. Together, this has resulted in the promotion of resistance in a variety of bacterial species, significantly reducing the value of tetracyclines as antibiotics [1, 3]. Increasing resistance has spawned the development of synthetically modified tetracyclines, such as minocycline, doxycycline and tigecycline; however, new forms of resistance encompassing the newer tetracyclines continue to emerge and spread [4].

Tetracycline resistance is often based on three main biochemical mechanisms, alone or in combination: (i) active export of the tetracycline molecules via efflux pumps, (ii) weakening of the interaction between tetracycline and the ribosome (ribosomal protection), and (iii) enzymatic degradation of tetracycline via hydroxylation [1, 4, 5]. Tetracycline resistance genes encoding these mechanisms are often encountered on mobile genetic elements, which makes them easily transferrable between bacteria. Indeed, the number of discovered and confirmed tetracycline resistance genes has increased dramatically over the last few decades, mainly due to horizontal gene transfer between bacteria [1]. To date, 62 different tetracycline resistance gene families have been discovered (<80 % amino acid sequence identity), where 35 encode efflux proteins, 13 encode ribosomal protection proteins and 14 encode inactivating enzymes [6].

Many antibiotic-resistance genes (ARGs) frequently encountered in clinical isolates are hypothesized not to originate from pathogenic bacteria. In fact, environmental and commensal bacteria maintain large and diverse resistomes from which ARGs can be mobilized into pathogens [7, 8]. A recent example is the gene *tet*(X), which was discovered in the 1980s and whose product catalyses the degradation of tetracyclines, including tigecycline [9–11]. During the last decade, *tet*(X) has been detected in a wide range of human pathogens, including *Klebsiella pneumoniae* and members of *Pseudomonadaceae *, and today constitutes an increasing clinical problem [12, 13]. The gene was first discovered on a transposable element in *Bacteroides fragilis *, a strict anaerobe, and given that *tet*(X) is dependent on oxygen, it has been suggested that this is not its original host [14]. Another example is the ribosomal protection gene *otr*(A), which has been found in *Streptomyces* spp. and *Mycobacterium* spp., but is expected to have an environmental origin [4]. This suggests that environmental bacteria harbour a collection – a reservoir – of tetracycline resistance genes that can be transferred into human pathogens over time [15]. The size and diversity of the tetracycline resistome are, however, unclear; therefore, we do not know which resistance genes may be transferred into pathogens in the future. Knowledge about the future forms of tetracycline resistance genes may enable early diagnostics and facilitate the implementation of management strategies to limit the spread of new forms of multiresistant bacteria.

Functional metagenomics – a method where random pieces of DNA from microbial communities are extracted and inserted into a bacterial host that is tested for induced phenotypes – is widely used to identify novel ARGs [16–18]. Analysis of soil using functional metagenomics was, for example, used to identify nine new genes for enzymatic degradation of tetracycline [*tet*(47)*–tet*(55)] [5]. Although functional metagenomics enables detection of novel ARGs without any prior knowledge about previously described genes, the method has a relatively low throughput (typically, ~10 Gb DNA is incorporated per experiment), which makes it unsuitable for characterization of novel ARGs at a larger scale. An alternative approach is to search for novel ARGs in genomic and shotgun metagenomic data. The great majority of newly discovered ARGs are evolutionarily related to resistance genes that are already known, allowing patterns of previously uncharacterized ARGs to be identified through sequence analysis. We have previously developed fARGene, a highly sensitive computational method based on hidden Markov models, for this particular purpose [19, 20]. By screening large volumes of genomic and metagenomic data we were able, for example, to significantly expand the number of subclass B1 metallo-β-lactamases, from 22 to 81 gene families [21]. Given the rapidly increasing volumes of bacterial genomic data present in public repositories, this approach has the potential to explore the diversity of novel ARGs in the bacterial communities at a much larger scale.

In this study, we aimed to expand the number of characterized tetracycline resistance genes and thereby provide a clearer picture of their evolutionary histories and origins. Through large-scale screening of genomic and metagenomic data using highly sensitive and optimized probabilistic gene models, we predicted 195 families of putative tetracycline resistance genes, of which 164 were novel and, thus, previously uncharacterized. Seventeen of the predicted genes were synthetically constructed and expressed in *Escherichia coli *, whereof seven resulted in an induced resistance phenotype. Phylogenetic analysis identified several horizontal gene transfer events between phyla. Our results are indicative that mobile efflux pumps were mobilized from *Proteobacteria *, while mobile ribosomal protection genes were mobilized from *Firmicutes* and/or *Actinobacteria *. The study presented here significantly expands the potential tetracycline resistome, and increases our knowledge about the diversity and evolutionary history of the tetracycline resistance genes.

## Methods

### Gene prediction using fARGene

The genes presented in this study were predicted using the recently developed computation method fARGene, which can reconstruct both known and novel genes from fragmented metagenomic data [19]. Briefly, the method starts by analysing raw reads using hmmer v.3.1b2 [22] and a hidden Markov model optimized for fragmented data. The reads classified as belonging to the gene class of interest are retrieved together with their read-pair and their quality is controlled using Trim Galore! v. 0.4.3 and a Phred score threshold of 30 [23]. The quality-controlled reads are assembled using SPAdes meta [24] and the assembled contigs then proceed to a second classification step where the threshold score is optimized for full-length genes. The contigs in which a gene of interest is predicted are retrieved and searched for ORFs using the National Center for Biotechnology Information (NCBI) ORFfinder (stand-alone version) [25]. The predicted ORFs are analysed with the model once again, in order to retrieve the ORFs with the highest score, and then passed as nucleotide and amino acid sequences as the final output. In addition to the metagenomic data, the method was also used to analyse genomic data, in which the assembly step is skipped and ORFs are predicted with Prodigal v2.6.3 [26] around the region where the gene of interest is identified. For full details, see our previous work [19].

### Model creation and optimization

For this study, three hidden Markov models were used. Each was built to represent a tetracycline resistance mechanism: efflux pumps of major facilitator superfamily (MFS) group 1, ribosomal protection and enzymatic degradation. Note that efflux pumps of other groups were not included in the study due to the limited number of known genes. The sensitivity for each model was optimized using leave-one-out cross-validation, while the specificity was optimized by applying the model to a set of closely related sequences that lacked the desired phenotype. The efflux model was built using 18 proteins belonging to the tetracycline efflux group 1 [1, 4, 6]. The specificity of the model was estimated using 192 proteins from the MFS [27, 28]. The ribosomal protection gene model was built of 11 confirmed tetracycline resistance ribosomal protection genes [6]. To estimate the specificity, 143 proteins belonging to conserved domain family elongation factor Tu GTP were used (PFAM00009). To build the enzyme model, all proteins verified as tetracycline resistance enzymatic genes [6] were downloaded and used to recreate a phylogenetic tree using ete npr v. 0.9.32 with parameters ‘-w standard_fasttree’ [29]. Based on the tree, the two genes *tet*(37) and *tet*(34) were removed due to their excessive diversion from the rest of the sequences. To enable the model to capture *tet*(X)-like genes, two variants of *tet*(X) were included in the model [*tet*(X) and *tetX3*]. As a negative dataset, 109 genes belonging to the conserved protein domain family UbiH (COG0654) were used. For a full list of genes used to build the three models see Table S1. The threshold scores for a fragment to be classified as positive were set based on manual inspection of the evaluation results, focusing on as high sensitivity as possible while still maintaining the highest possible specificity, with a minimum limit of 0.9. For full-length genes, the threshold scores were set so that both sensitivity and specificity were one for all models except for efflux pumps. For the efflux pumps, the gene *tet*(42) had a significantly lower score compared to the other genes in the cross-validation, and the resulting threshold was set so that all efflux pump genes except *tet*(42) would be captured by the model, resulting in a sensitivity of 0.94. The resulting sensitivity and specificity for the three models can be seen in Table S2.

### Post-processing and phylogenetic analysis

The predicted genes were grouped based on their resistance mechanisms and then clustered at 100 % sequence identity using usearch v. 8.0.1445 [30], parameters ‘-cluster-fast -id 1’, to remove redundant genes. Then, the genes were clustered together with all previously known tetracycline resistance genes with the same resistance mechanism with a 70 % amino acid sequence identity using usearch with parameters ‘-cluster-fast -id 0.7’. The 70 % sequence identity cut-off was chosen to ensure that previously known genes clustered consistently and, thus, that no gene family would incorrectly be annotated as new. The clusters that did not contain any previously known gene were called ‘novel family’ (NF) and the number of genes clustering in any of the novel families were counted as well as the number of families. The generated centroid files from the clustering were then aligned using mafft v. 7.273 [31] and phylogenetic trees were recreated using FastTree v. 2.1.10 with default settings [32]. To perform the phylum analysis, the bacterial species of each genome from NCBI RefSeq and Human Microbiome Project (HMP) genomic databases carrying at least one tetracycline resistance gene were extracted and counted. Duplicated species were removed and those remaining were organized into groups based on their phyla. The number of unique species in a phylum carrying a tetracycline resistance gene was then compared to the total number of unique species in this phylum in the original dataset using Fisher’s exact test.

### Genetic context analysis

All genomes available in the GenBank assembly database, as well as all GenBank plasmid sequences (downloaded January 2019) were searched for the novel gene family amino acid sequences using diamond v0.9.24.125 blastx with an identity cut-off of 70 % [33]. For every hit, up to 20 kb upstream and downstream were extracted and annotated using prokka v1.12 [34]. Sequences annotated as hypothetical proteins were searched against the UniprotKB database using diamond blastx with a 50 % identity cut-off to investigate their potential function. The genetic environments of selected genes were further searched for mobile genetic elements using ISFinder and CDD search [35, 36].

### Experimental validation

Potential tetracycline resistance genes were synthesized by Thermo Fisher Scientific, using the GeneArt gene synthesis service and provided in the vector pMK. Each candidate gene sequence was equipped with the promoter P*_bla_* plus ribosomal binding site upstream and the *rrnB* terminator T1 downstream to ensure constitutive expression. The negative control consisted of the expression elements without any gene, the positive controls were *tet(X)* [37] and *tet(A)* [38]. The pMK plasmids containing the resistance gene candidates were used to transform chemically competent *E. coli* TOP10 (Invitrogen, Thermo Fisher Scientific). The functionality of candidate genes was verified against both tetracycline (Sigma Aldrich, Merck) and tigecycline (Glentham Life Sciences). Minimum inhibitory concentrations (MICs) were determined by broth microdilution in cation-adjusted Mueller-Hinton medium. In accordance with European Committee on Antimicrobial Susceptibility Testing (EUCAST) guidelines (clinical breakpoint tables v. 9.0), medium for tigecycline tests was prepared freshly on the day of use. Serial dilutions of the tested antibiotics were prepared in triplicate in 96-well plates and inoculated with 5×10^5^ cells ml^−1^ in each well [39] at a final volume of 200 µl. After 24 h incubation at 37 °C and 180 r.p.m., optical density (OD_650_) was measured (Spectramax 340PC 384; Molecular Devices) and the MIC was defined as the lowest concentration of an antimicrobial that reduced growth to an OD_650_ ≤0.2 [40].

**Table 2.** Summary of the functional verification in *E. coli* of 17 predicted genes

Mechanism	Gene ID	Gene family	Fold-change tetracycline MIC	Increased growth rate*	Species	Most similar previously known	Amino acid identity to most similar known (%)
Enzyme	G8	NF3	–	nd	* Labilithrix luteola *	*tet*(55)	40.3
Enzyme	G35	NF11	–	nd	* Bacillus simplex *	*tet*(55)	39.7
Enzyme	G38	NF13	–	nd	* Legionella lansingensis *	*tet*(49)	41.5
Enzyme	G42	NF17	128	nd	Metagenome	*tet*(47)	54.0
Enzyme	G47	NF20	–	nd	* Niabella ginsenosidivorans *	*tet*(X)	63.9
Ribosomal protection	G139	NF28	–	nd	* Jiangella alkaliphila *	*otr*(A)	52.0
Ribosomal protection	G161	NF35	–	nd	* Cloacibacillus porcorum *	*tetB*(P)	34.7
Ribosomal protection	G231	NF54	1–4	p=1.6×10^−4^	* Ensifer adhaerens* (P)†	*otr*(A)	44.5
Ribosomal protection	G242	NF55	1–4	p=5.8×10^−3^	* Pseudomonas chlororaphis *	*otr*(A)	43.9
Ribosomal protection	G307	NF60	16	nd	* Clostridium saccharolyticum *	*tet*(44)	51.4
Ribosomal protection	G404	NF81	–	nd	* Rhizobium* sp. LPU83 (P)†	*otr*(A)	44.7
Efflux	G131	NF12	–	nd	* Burkholderia* sp. PAMC26561 (P)†	*tet*(A)	49.7
Efflux	G160	NF17	–	nd	* Ensifer adhaerens* (P)†	*tet*(41)	50.7
Efflux	G241	NF21	–	p=0.26	* Acinetobacter baumannii *	*tet*(41)	44.6
Efflux	G256	NF26	256–512	nd	Uncultured bacterium	*tet*(G)	62.8
Efflux	G281	NF34	128–256	nd	* Ochrobactrum anthropi *	*tet*(G)	55.3
Efflux	G296	Tet(A)/Tet(C)	2–16	nd	* Achromobacter xylosoxidans *	*tet*(A)	76.0

nd, Not determined.

*P values calculated at hour 24 with a tetracycline concentration of 0.45 µg ml^−1^.

†Plasmid.

Growth behaviour of selected constructs was determined at different tetracycline concentrations using the OmniLog system (Biolog). Bacteria were grown in 96-well plates as described above with the addition of redox dye A. During 24 h incubation at 37 °C, metabolic activity was measured every 15 min in the form of a colour change caused by the reduction of the dye (OmniLog units). Growth curves and standard deviations were calculated from the mean of three independent experiments, each consisting of three technical replicates. The reported *P* values were calculated using a one-sided two-sample *t*-test with the values taken from the 24th hour of incubation at a tetracycline concentration of 0.45 µg ml^−1^. Since the current nomenclature of tetracycline resistance genes requires that the putative gene should be shown to be functional in its original host, none of the experimentally verified genes could be given an official name.

## Results

### Creation and evaluation of tetracycline resistance gene models

Genomic and metagenomic data were analysed for tetracycline resistance genes (both known and new) using fARGene, a computational method that applies probabilistic gene models to find ARGs (see our previously published work [19]). Three gene models were developed, corresponding to each of the three major mechanisms of tetracycline resistance: the MFS efflux pumps of type group 1, ribosomal protection genes and enzymatic degradation genes [1]. The models were trained using previously known tetracycline resistance genes, and the sensitivity and specificity were optimized for both short reads and full-length genes using cross-validation. The resulting models all had a specificity of 1 for full-length genes, while the full-length sensitivity was 1 for all but the efflux pump model, which had a sensitivity of 0.94. The specificity for fragments (100 nucleotides) was at least 0.90 for all models (0.93, 0.90 and 0.97 for enzymatic, ribosomal protection and efflux pump genes, respectively), while the sensitivity ranged from 0.78 (ribosomal protection genes) to 0.96 (enzymatic degradation genes) (see Methods and Table S2 for more details). Using the gene models, fARGene was applied to more than 12 Tb of genomic and fragmented metagenomic data. This resulted in 1354 unique predicted tetracycline resistance genes, which were clustered into 195 gene families, of which 164 families were novel (amino acid sequence identity <70 % to any previously known tetracycline resistance gene) (Tables 1 and S3). Genes encoding ribosomal protection genes were most common (927 genes in 116 families, of which 106 were novel), followed by efflux genes (331 genes in 53 families, of which 38 were novel) and enzymatic degradation genes (96 genes in 26 families, of which 20 were novel).

### Prediction of tetracycline-resistance genes in genome sequence data

**Fig. 1.**
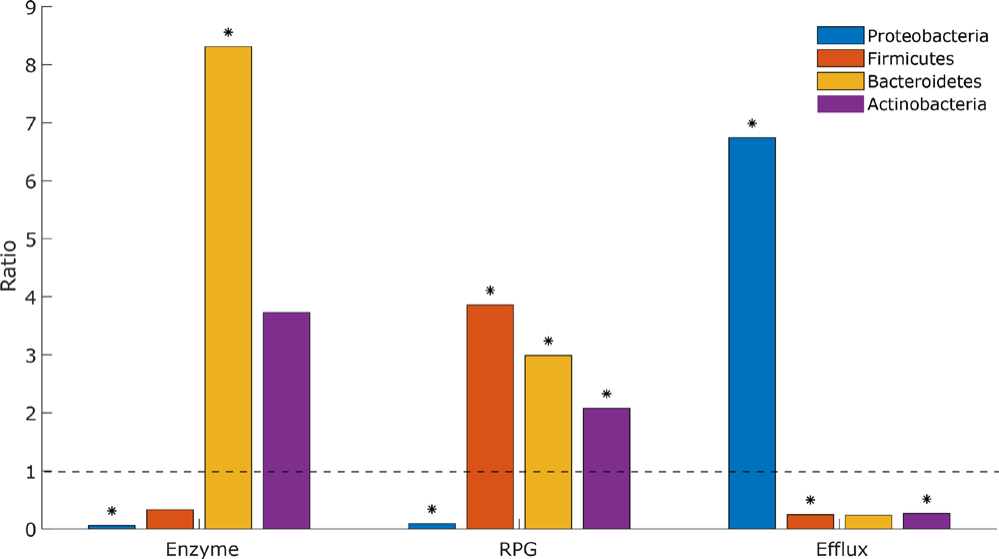
Phylum analysis of genomes carrying tetracycline resistance genes. The analysis was based on genomes present in NCBI RefSeq and HMP genomic databases. The significance of the ratios was assessed using Fisher’s exact test and results with *P* <0.001 are marked by asterisks. RPG, Ribosomal protection genes.

The genomic data (NCBI genome, NCBI plasmids, NCBI nt, NCBI environmental, HMP genomic [41]) yielded a prediction of 1278 unique tetracycline resistance genes (Table 1). Among the 7376 complete genomes present in the NCBI genome database, 542 (7.3 %) carried at least one predicted tetracycline-resistance gene, whereof 51 (0.7 %) and 3 (0.04 %) carried two and three genes, respectively. Ribosomal protection genes were most commonly predicted (314 genomes, 4.3 %), followed by efflux pumps (244 genomes, 3.3 %) and degradation enzymes (32 genomes, 0.4 %). In addition, 458 (4.0 %) plasmids present in the NCBI plasmids database carried at least one predicted tetracycline resistance gene (Table 1). Enrichment analysis, where the observed proportion of predicted resistant species in each phylum in NCBI genome (7376 genomes) and HMP genomic (1271 genomes) was compared to what was expected by chance, showed that the distribution of tetracycline-resistance genes was associated with taxonomy (see Methods). In particular, *Proteobacteria* were underrepresented carriers of both ribosomal protection genes (ratio 0.09 with *P* <10^−15^) and enzymatic degradation genes (ratio 0.06, *P*=1.4×10^−4^), while efflux pump genes were overrepresented (ratio 6.74, *P* <10^−15^) (Fig. 1). In contrast, *Firmicutes* showed an overrepresentation of ribosomal protection genes (ratio 3.86, *P* <10^−15^), while *Bacteroidetes* showed an overrepresentation of both ribosomal protection and enzymatic degradation genes (ratio 2.99, *P*=5.8×10^−11^, and ratio 8.31, *P*=9.0×10^−6^, respectively). Finally, *Actinobacteria* showed an overrepresentation of ribosomal protection genes and an underrepresentation of efflux pump genes (ratio 2.08, *P*=5.3×10^−7^, and ratio 0.27, *P*=2.3×10^−4^, respectively).

### Prediction of tetracycline resistance genes in metagenomic data

**Fig. 2.**
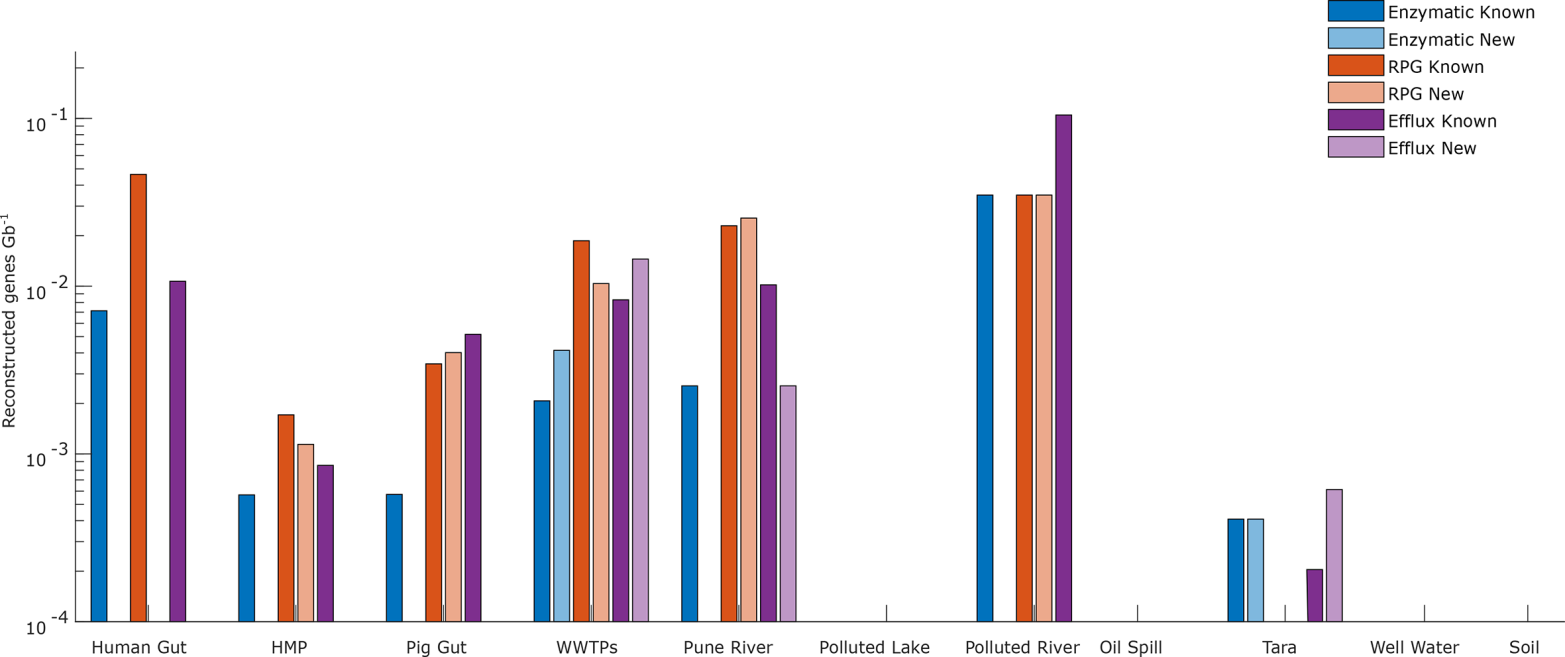
Number of reconstructed genes Gb^−1^ for each metagenomic dataset. A reconstructed gene was classified as novel if it had a sequence identity of <70 % to any previously known tetracycline resistance gene. RPG, Ribosomal protection genes.

From the metagenomic data, which consisted of human and animal-related sources, as well as samples from polluted and more pristine environments (Table 1), 98 non-redundant genes (123 genes in total) were predicted. Similar to the genomic data, ribosomal protection genes were the most common (61 unique genes corresponding to 24 families, of which 17 were novel), followed by efflux pumps (28 genes in 16 families, of which 7 were novel) and enzymatic degradation genes (9 genes in 8 families, of which 5 were novel) (Fig. 2). This corresponded to 0.006, 0.0024 and 0.0012 reconstructed genes Gb^−1^ for ribosomal protection genes, efflux pumps and degradation enzymes, respectively. Putative tetracycline resistance genes were found in all but four of the metagenomic datasets, but their abundance varied considerably between communities. The highest number of genes was found in the metagenomes from Swedish wastewater treatment plants (WWTPs) [42], sediments from Pune river (Pune, India) [43] and in pig microbiome (pig gut) [44]. Furthermore, the wastewater treatment plant metagenome was the only dataset that contained novel genes of all three resistance mechanisms. Efflux pump genes were especially abundant in wastewater treatment plants and the pig microbiome.

Ribosomal protection genes were only found in the human and animal-related datasets together with sediments from two polluted rivers (human gut, HMP metagenome, WWTPs, pig gut, Pune river and polluted river (Isakavagu, India)) [45–47]. Compared to efflux pump genes and enzymatic degradation genes, ribosomal protection genes were highly abundant in the human and pig microbiomes. Furthermore, only 4 out of 23 (17 %) ribosomal protections genes found in the human microbiome (human gut and HMP metagenome) were novel. The human gut metagenome (human gut) only contained previously known genes, while the HMP metagenome did neither contain any novel enzymatic nor novel efflux pump genes. Enzymatic degradation genes were rare overall, but had the largest proportion of novel genes (5 out of 9, 56 %), which were all found in the wastewater treatment plant and marine environment (WWTPs and Tara Ocean [48]) metagenomes. The previously known tetracycline resistance genes found in the most environments were *tet*(A) (efflux), *tetB*(P) (ribosomal) and *tet*(X) (enzymatic), which all were found in five environments, respectively (Table S4). Finally, the analysis of data from oil-contaminated deep-sea water (oil spill) [49], the Kazipally lake in India polluted with wastewater from pharmaceutical manufacturing (polluted lake) [50], and bacterial communities in Indian soil and well water [51] did not yield any reconstructed full-length tetracycline resistance genes.

### Experimental verification

Next, we investigated whether some of the predicted genes induced a resistance phenotype in *E. coli *. In total, 17 predicted novel genes – 5 enzymatic degradation genes, 6 ribosomal protection genes and 6 efflux genes – were chosen from different parts of their respective phylogenetic tree. The genes were synthesized and inserted into an *E. coli* host, after which the MIC was determined (Table 2, Methods). For three genes, the growth rate in the presence of tetracycline was also measured (G231 in NF54 and G242 in NF55 ribosomal protection; G241 in NF21 efflux). For seven of the tested genes, expression in *E. coli* resulted in a resistance phenotype (Table 2). The highest increase in MIC was >256-fold and was observed for the efflux gene G256 in NF26. MIC assays were inconclusive for two of the ribosomal protection genes (increase in MIC between 1 and 4), but a more detailed analysis of growth rate over time showed that both genes increased growth significantly compared to the control (Fig. S1). Ten genes did not induce a resistance phenotype in *E. coli* (Table 2). Non-functionality in the tested host is, however, not evidence for lack of resistance function, as the genes may be functional in their native context and species more closely related to their actual hosts. Even in *E. coli *, the resulting MIC for a given ARG can be highly strain-dependent [52]; hence, we put more emphasis on increases in MICs rather than on the absolute MIC values.

### Phylogenetic analysis

**Fig. 3.**
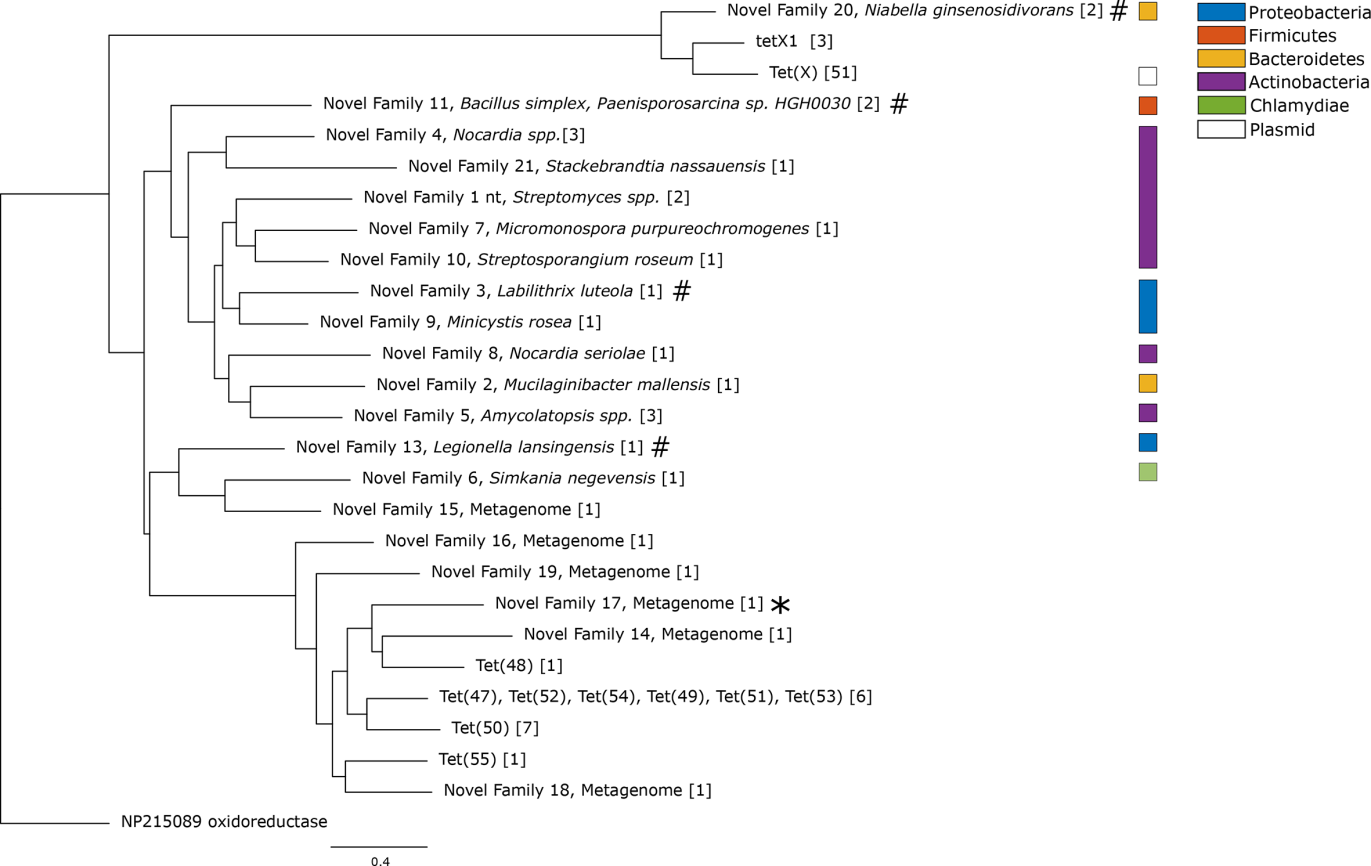
Phylogenetic tree of the enzymatic tetracycline-resistance genes predicted in this study. The tree was recreated from all previously known enzymatic tetracycline-resistance gene families, together with gene families predicted in this study. Each gene family contains genes with >70 % amino acid sequence identity, and the number of unique genes in each gene family is presented within the square brackets. The gene functional in *E. coli* is indicated by an asterisk and the gene families for which the tested genes did not function in *E. coli* are indicated by hash signs. The scale bar indicates number of substitutions per site.

Next, all predicted tetracycline resistance genes (1354 unique genes, corresponding to 195 families) were used to recreate three phylogenetic trees, one for each resistance mechanism (see Methods). The phylogenetic tree of the enzymatic degradation genes, consisting of 26 gene families (Figs 3 and S2), was separated into two monophyletic groups. The first group consisted of the known *tet*(X), a full-length variant of the *tet*(X) homologue *tetX1* [53] and a novel gene family (NF20). In addition to the predicted novel genes, we investigated whether the full-length variant of *tetX1* induced a resistance phenotype in *E. coli *. However, similar to previous reports on the truncated variant of *tetX1*, we did not see any increase in MIC against either tetracycline or tigecycline for either NF20 (G47) or the full-length *tetX1* variant [10]. The second group contained all other previously known enzymatic degradation genes [*tet*(47) to *tet*(55)] and was more diverse, with genes from five phyla and a wide range of metagenomic datasets (Fig. 3). Interestingly, the clade where *tet*(47) to *tet*(55) were located contained only two genes found in genomes, which were both pathogens: the proteobacterial species *Legionella lansingensis* and the *Chlamydiae* species *Simkania negevensis* [54, 55].

**Fig. 4.**
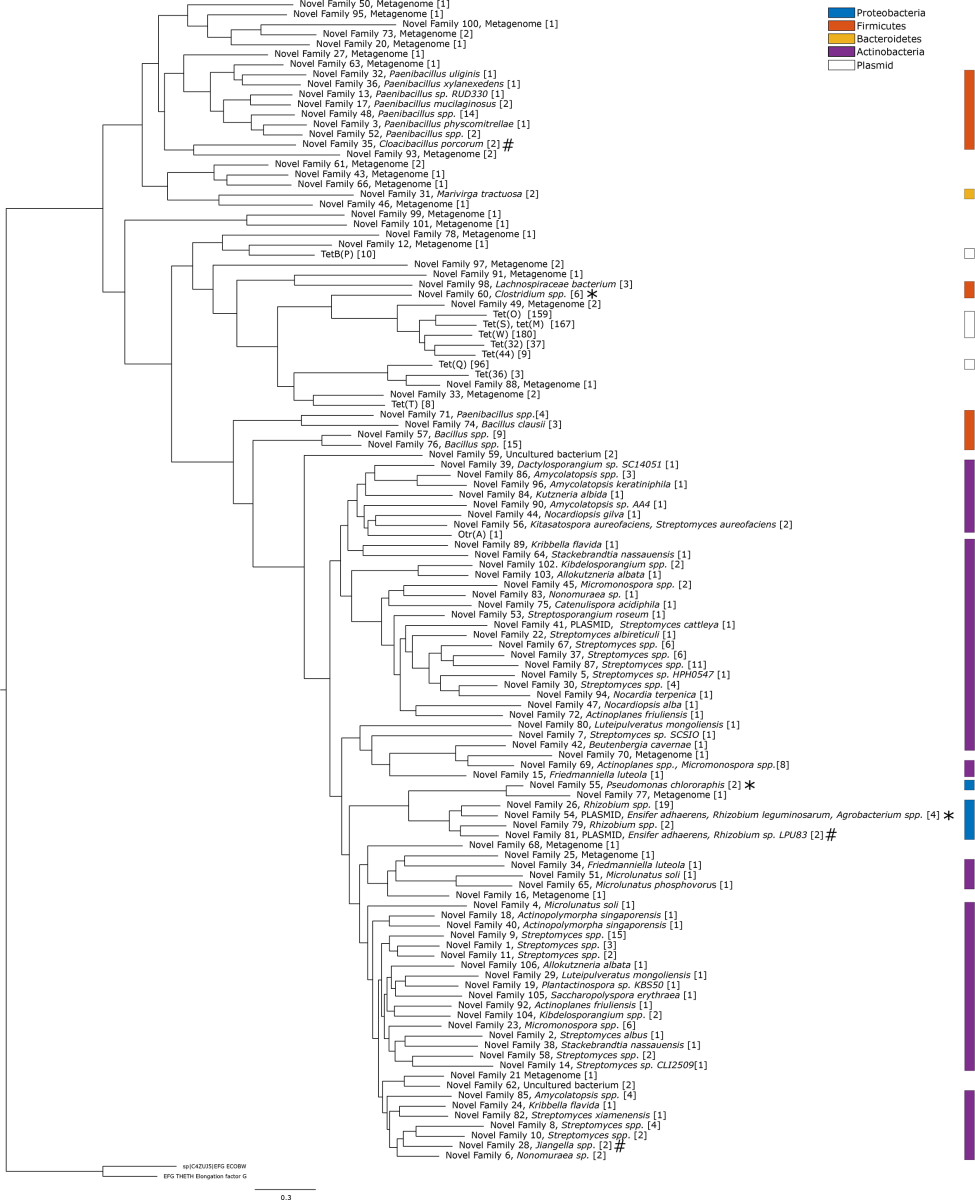
Phylogenetic tree of the ribosomal protection genes predicted in this study. The tree was recreated from all previously known ribosomal protection gene families, together with gene families predicted in this study. Each gene family contains genes with >70 % amino acid sequence identity, and the number of unique genes in each family is presented within the square brackets. The gene families functional in *E. coli* are indicated by asterisks and the gene families for which the tested genes did not function in *E. coli* are indicated by hash signs. The scale bar indicates number of substitutions per site.

The phylogenetic analysis of the ribosomal protection genes was based on a total of 116 gene families (Figs 4 and S3). Similarly to the enzymatic degradation genes, the tree could be divided into two major groups. The first group contained all previously known ribosomal protection genes except for *otr*(A). The first group could be further separated into two distinct clades. Clade one in group one contained all of the previously known genes together with genes reconstructed from the bacterial communities from river sediment (Pune river) and wastewater treatment plants (WWTPs). The second clade in group one contained only novel genes, of which several were found in the pig gut metagenome. Relatively few of the genes in the first group were identified in genomic data, and this could not be associated with any host. However, two gene families that were the closest related to the previously known genes were both found in *Firmicutes* species: NF60, identified in *Clostridium saccharolyticum* and the pathogen *Clostridium sphenoides* [56]; and NF98, identified in *Lanchospiraceae* sp. The NF60 gene variant G307 in *Clostridium saccharolyticum* was functional when expressed in *E. coli *. Genes from NF60 were also commonly detected in metagenomes from both the environment and the human microbiome, suggesting that these genes are widely spread. Furthermore, several species from *Paenibacillus* – a genus responsible for the American foulbrood disease in honeybees and commonly treated with tetracycline – carried predicted novel families of ribosomal protection genes [57]. The second group of the phylogenetic tree contained the known *otr*(A) gene, which was first discovered in an oxytetracycline-producing *Streptomyces* spp. [4, 58, 59]. This part of the tree consisted mainly of genes located in other *Actinobacteria* (56 novel families). A small clade within the second group with novel gene families from proteobacterial species contained two genes located on plasmids (G232 in NF54, *Ensifer adhaerens *, and G404 in NF81, *Rhizobium* sp. LPU83) indicative of horizontal gene transfer across bacterial phyla. In the same clade, another resistance gene was present in *Pseudomonas chlororaphis *. This species is root-colonizing and has previously been shown to promote plant growth and to have biopesticide properties [60]. When expressed in *E. coli *, the gene located on the plasmid in *Ensifer adhaerens* and the one located in *Pseudomonas chlororaphis* did not result in an increased MIC, but both showed significantly increased growth rates in the presence of tetracycline compared to the negative control (Table 2, Fig. S1).

**Fig. 5.**
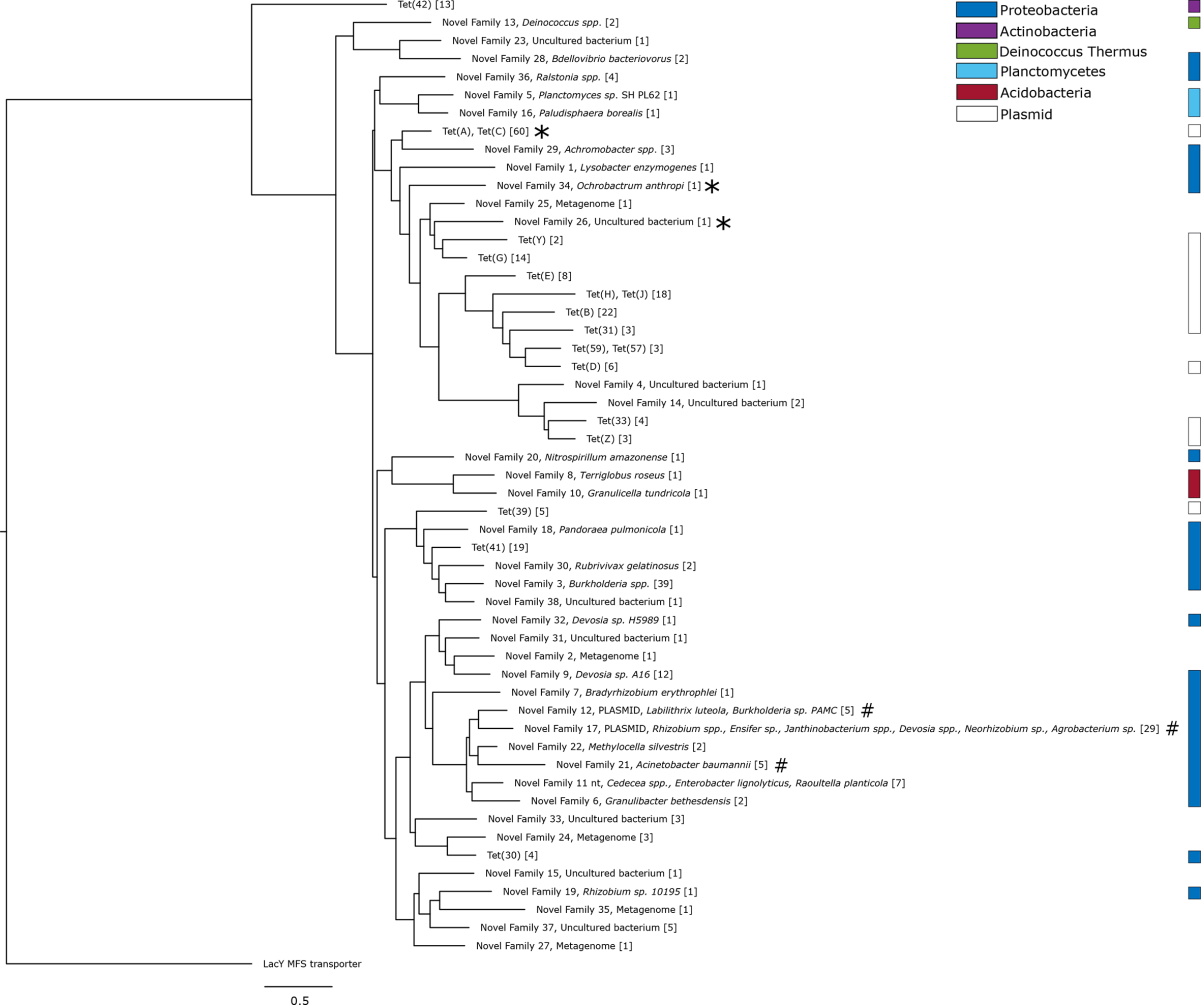
Phylogenetic tree of the efflux pump genes predicted in this study. The tree describes the previously known efflux gene families of MFS group 1, together with gene families predicted in this study. Each gene family contains genes with >70 % amino acid sequence identity, and the number of unique genes in each group is presented within the square brackets. The gene families functional in *E. coli* are indicated by asterisks and the gene families for which the tested genes did not function in *E. coli* are indicated by hash signs. The scale bar indicates number of substitutions per site.

The phylogenetic tree of the efflux pump genes consisted mainly of genes found in genomic data. Most genes appeared in *Proteobacteria *, but some were also found in species of *Acidobacteria *, *Actinobacteria *, *Planctomycetes* and *Deinococcus–Thermus* (Figs 5 and S4). The known efflux pump genes were distributed over the entire tree, but several accumulated in a clade containing novel genes from *Alphaproteobacteria* and *Betaproteobacteria* (including the pathogens *Ochrobactrum anthropi* and *Achromobacter* spp.). Furthermore, we identified two novel families (NF12, NF17) with genes located on plasmids. The plasmid carrying a variant of NF17 was found in *Ensifer adhaerens *, but located on a different plasmid separate from the ribosomal protection gene present in the same species. The gene family NF17 also contained genes from five additional genera of *Alphaproteobacteria *: *Rhizobium *, *Janthinobacterium *, *Devosia *, *Agrobacterium* and *Neorhizobium *. Finally, a plasmid harbouring NF12 was found in *Burkholderia* sp. PAMC 26561 (G131), while other members of the same gene family were found in *Labilithrix luteola* and the marine metagenome (Tara Ocean).

## Discussion

In this study, we have presented a large-scale prediction of novel tetracycline resistance genes. This was performed by using sensitive probabilistic models developed to specifically identify novel tetracycline resistance genes, even when they have low sequence identity to known resistance genes [19]. Large amounts of genomic and metagenomic data were screened, and the predicted genes were used to infer their evolutionary history. Our study identified all previously known tetracycline resistance genes of the three modelled main mechanisms, together with 164 predicted novel gene families. These results expand the potential tetracycline resistome significantly. In addition, experimental validation of 17 predicted genes showed that 7 of these resulted in a resistance phenotype when expressed in *E. coli *, indicating that they are functional in this host.

The number of predicted genes differed considerably between the three main resistance mechanisms investigated in this study: efflux pump genes, ribosomal protection genes and enzyme degradation genes. Most common were ribosomal protection genes (927), followed by efflux pumps genes (331) and enzymatic degradation genes (96). Efflux pump genes were found in a larger number of phyla (nine), compared to six and five phyla for ribosomal protection genes and enzymatic degradation genes, respectively. There were also clear taxonomic differences between genes from the three resistance mechanisms. Efflux pump genes were overrepresented in *Proteobacteria* with more than 8 % of the unique proteobacterial species in HMP genomic and NCBI RefSeq carrying an efflux pump gene, while enzymatic degradation and ribosomal protection genes were underrepresented in *Proteobacteria *. These resistance mechanisms were more common in other phyla, in particular *Bacteroidetes *, and for ribosomal protection genes, also *Actinobacteria* and *Firmicutes *. In contrast, only three species of *Bacteroidetes* were carrying efflux pump genes. Note, however, that the enzymatic degradation genes found in *Bacteroidetes* belonged to only four gene families, which included *tet*(X), *tetX1* and novel family 20 that is closely related to *tet*(X) and *tet*X1. A similar pattern could be seen for the ribosomal protection genes, where all genes located in *Bacteroidetes* were either previously known or from a single novel family (NF31). Thus, even though enzymatic degradation genes and ribosomal protection genes were common in species from *Bacteroidetes *, these numbers represent mainly known mobile genes and, thus, only a limited part of the full diversity of these gene classes. However, it should be pointed out that the genomic data used in this study is likely biased towards well-studied organisms, in particular those related to human health; thus, the results are not representative of the full diversity of the tetracycline resistome.

The relative number of reconstructed tetracycline resistance genes varied between environments. The human and pig microbiomes, as well as environments that are potentially polluted by human faecal matter, all contained high levels of genes from all three resistance mechanisms. In these environments, the enzymatic genes were heavily dominated by *tet*(X) and the previously studied *tet*(X)-like gene *tetX1* [10, 50]. A similar pattern was observed for ribosomal protection and efflux pumps genes, where the human microbiome contained almost exclusively previously known genes. Here, the abundance was especially high in the intestinal tract where *Bacteroidetes* is dominant [45], adding additional support to the hypothesis that bacteria from this phylum seem to carry a relatively limited set of mostly known tetracycline resistance genes. The environments that are likely to contain less faecal matter contained no tetracycline resistance genes at all, except for marine microbial communities in the Tara Ocean dataset, which contained both known and novel efflux pumps and enzymatic genes. An environment with a high abundance and diversity of novel resistance genes may indicate a higher risk for transfer events into recipients that will enable spread to pathogens. Based on our analysis, such environments include the human and animal-associated microbiomes, as well as the bacterial communities from the wastewater treatment plant (Table 1). Note, however, that aside from the Tara Ocean data, the environmental datasets used in this study were also the least deeply sequenced (especially the polluted lake dataset) and considering that the abundance needs to be fairly high for a gene to be reconstructed from a metagenomic dataset, it is likely that some resistance genes were below the limit of detection [61, 62]. Thus, we cannot exclude that there is a much larger diversity of novel tetracycline resistance genes in these communities than what is possible to detect with existing data.

The phylogenetic analysis showed the presence of potentially mobile novel tetracycline resistance genes. For example, the phylogenetic group containing the known ribosomal protection gene *otr*(A), which was dominated by *Actinobacteria *, contained a small clade of gene families from proteobacterial species (Fig. 4). One of these gene families (NF81) comprised a plasmid-borne gene from *Rhizobium* sp*.* LPU83 (G404), while another gene family (NF54) harboured a gene on a plasmid from *Ensifer adhaerens* (G231). Other members of this family are located in the genomes of *Rhizobium* sp. and *Agrobacterium* spp. None of these genes showed a consistent increase in MIC when expressed in *E. coli *. However, the gene located on the *Ensifer adhaerens* plasmid was tested in the growth assay and allowed significantly faster growth than the control, indicating that it is indeed functional. One additional gene family (NF55) from the same clade was found in *Pseudomonas chlororaphis *, and was also shown to induce a resistance phenotype in *E. coli *. The members of the genus *Pseudomonas* are known to have highly plastic genomes and to commonly harbour conjugative elements carrying ARGs [63]. Interestingly, the gene was located on a ~13 kb long insert present in three *Pseudomonas chlororaphis* strains, but absent in three other strains and all other *Pseudomonas* species. This insert also contained distant homologues to fosfomycin-resistance genes (33.6 % amino acid identity to FosA) and macrolides (34.9 % amino acid identity to OleC). Taken together, this indicates that there has been a horizontal gene transfer event of ribosomal protection genes from species within *Actinobacteria* to *Proteobacteria *. Since the genes are present on mobile genetic elements or, as in the case of *Pseudomonas chlororaphis *, highly plastic genomic regions, there is an apparent risk that they can spread further, including to human pathogens.

There were also indications of new mobile efflux pump genes. For example, two of the novel gene families had genes located on plasmids, one in *Burkholderia* sp. PAMC 26561 (G131 in NF12) and one in *Ensifer adhaerens* (G160 in NF17), although not the same strain as the one carrying a predicted ribosomal protection gene. Furthermore, genes within NF17 were found in the genomes of *Janthinobacterium* spp. and several species within the order *Rhizobiales* (* Agrobacterium* sp., *Rhizobium* spp., *Devosia* spp. and *Neorhizobium* sp.). Many of these species are symbiotic nitrogen-fixating bacteria associated with root nodules, and are known to often carry plasmids or megaplasmids harbouring genes essential for symbiosis with the host plant [64, 65]. Functional verification showed, however, that these genes did not result in an increased MIC when expressed in *E. coli *. Moreover, several of the predicted new efflux pump genes were located in opportunistic or pathogenic bacteria, e.g. *Acinetobacter baumannii *, *Achromobacter insolitus *, *O. anthropi *, *Pandoraea pulmonicola* and *Burkholderia cenocepacia* [66–70]. Among these, *Acinetobacter baumannii* is a notorious pathogen harbouring a wide range of mobile resistance genes against clinically relevant antibiotics, including tetracyclines [71]. The gene family identified in *Acinetobacter baumannii* was only present in 25 of 155 *Acinetobacter baumannii* strains in NCBI RefSeq. However, when a member of this gene family (G241) was expressed in *E. coli *, we did not observe any increase in MIC. Closer analysis of the regions surrounding the identified gene in the 25 strains of *Acinetobacter baumannii* revealed an insertion sequence (IS*Aba6*) located in direct connection to the gene in one of the strains, suggesting that the gene could potentially be mobile [72].

Analysis of the phylogenetic tree in the neighbourhood around the previously known mobile tetracycline resistance genes provided further insights into their recent evolutionary history. Phylogenetic analysis of the ribosomal protection genes showed, for example, that *otr*(A) was most closely related to genes found in various *Actinobacteria *, including many species from the genus *Streptomyces* (Fig. 4). The chromosomal gene closest to *otr*(A) was located in the species *Amycolatopsis* sp., but the evolutionary distance to *otr*(A) was still substantial. The *otr*(A) gene has previously been found in *Streptomyces* spp. and *Mycobacterium* spp. [4]. and our results suggest, in accordance with the literature, that this gene has likely been mobilized from an unknown *Actinobacteria* species [4, 73]. Indeed, the majority of the clinically used antibiotics are produced by *Actinobacteria *, and several classes of resistance genes are hypothesized to originate from this phylum [74, 75]. The other known mobile ribosomal protection genes were located in a single clade where only two of the novel gene families were found in isolated species, both *Firmicutes* (NF98, *Lachnospiraceae* spp., and NF60, *Clostridium* spp.). Ribosomal protection genes were in general common in *Bacteroidetes *, but no species in this phylum contained a chromosomal non-mobile gene that was close evolutionary to any mobile ribosomal protection gene. Therefore, it is plausible that the mobile ribosomal protection genes originated elsewhere – based on the current evidence most likely *Firmicutes* – and were horizontally transferred to *Bacteroidetes *. It should be pointed out that a wide range of species from both phyla are naturally occurring in animal and human microbiomes, suggesting that there is both ecological connectivity and tetracycline selection pressure that may enable horizontal gene transfer.

The phylogenetic tree of the efflux pump genes (Fig. 5) was heavily dominated by genes from *Proteobacteria* and all of the known mobile efflux pumps had their closest relative within this phylum. This strongly suggests that mobile efflux pump genes were mobilized from *Proteobacteria *. However, the evolutionary distances were large and no mobile efflux pump gene with high sequence identity to a non-mobile efflux pump gene was found, suggesting that the origin – or origins – are species with yet uncharacterized genomes. Note, however, that one of the genes closest related to any previously known mobile efflux pump gene was located in *O. anthropi* (* Betaproteobacteria *), an opportunistic pathogen known to thrive in a large variety of habitats [76]. Furthermore, the *O. anthropi* gene showed high resistance against tetracycline when expressed in *E. coli *.

The enzymatic resistance genes had few matches in the genomic data, and based on the available information, it is difficult to assess their origin with certainty. We did, however, identify a new family related to *tet*(X) (NF20), which, like the *tetX1* and the *tet*(X) family, was found in *Bacteroidetes* species (Fig. 3). Furthermore, the *tetX1* genes were always found together with a copy of *tet*(X), except for a reconstructed *tetX1* gene from the pig microbiome. Interestingly, this was not the case for the genes in NF20, which appeared both in *Niabella ginsenosidivorans* and in a metagenome from a wastewater treatment plant. We were not able to identify any elements associated with known mechanisms for horizontal gene transfer, suggesting that these genes may be chromosomal. Considering the high degree of mobility among *tet*(X)-like genes in *Bacteroidetes* [12], it is hard to assess if this is the origin of the gene or if the genes were horizontally transferred to this part of the taxonomic tree.

The method, fARGene, used in this study applies probabilistic gene models in the form of hidden Markov models to identify novel resistance genes [22]. The models have been optimized to accurately discriminate between homologues with and without a resistance phenotype to keep the number of false positives low [19]. Nevertheless, all genes identified in this study are predictions and, therefore, it is necessary to experimentally verify if they can induce resistance phenotypes and, if that is the case, in what hosts they are functional. In this study, we selected 17 of the predicted genes for verification in *E. coli* and among those 7 were found to be functional. Note, however, that even some well-known tetracycline resistance genes that are functional in other species do not induce a significant resistance phenotype in *E. coli *. In particular, ribosomal protection genes, which are rare in *Proteobacteria *, confer only low levels of resistance when expressed in *E. coli* [4]. Indeed, several ribosomal protection genes showed only a small increase in MIC, but the functionality of two of these genes could be verified with the considerably more sensitive growth assay. Therefore, it cannot be excluded that other genes which did not result in an increased MIC in *E. coli* may be functional and, potentially, be much more potent in other hosts. In addition, experimental verification was performed based on the exact predicted protein sequences without codon optimization; therefore, an impaired expression may explain the lack of function in *E. coli *. Furthermore, we cannot exclude that some of the predicted genes may have other molecules as their natural substrate and, thus, demonstrate a lower efficacy for tetracyclines. Also, we cannot disregard the possibility that some of the predicted genes may be false positives; thus, they should be considered putative until further experimental verification has been made.

### Conclusion

Screening of more than 12 Tb of genomic and metagenomic data was used to identify 195 families of tetracycline resistance genes, of which 164 families were novel predictions and previously uncharacterized. Phylogenetic analysis suggested that mobile tetracycline resistance genes were mobilized from different parts of the taxonomical tree, where ribosomal protection genes originated from *Firmicutes* and *Actinobacteria *, while efflux pump genes originated from proteobacterial species. Enzymatic genes were scarce; therefore, their evolutionary origin remains unclear. Several of the novel mobile genes showed patterns of horizontal transfer between bacterial phyla. This study expands the known tetracycline resistome, and describes the reservoir of resistant determinants maintained by environmental and commensal bacteria. If mobilized and transferred to pathogens, these genes could reduce the efficacy of existing and future tetracycline antibiotics and thereby pose a significant threat to our ability to treat bacterial infections.

## Supplementary Data

Supplementary material 1

Supplementary material 2
