# Supplementary material for: Comprehensive screening of genomic and metagenomic data reveals a large diversity of tetracycline resistance genes
Source: Microb Genom. 2020 Oct 30;6(11):mgen000455. doi: 10.1099/mgen.0.000455 (PMC7725328; doi:10.1099/mgen.0.000455)
Supplement: Supplementary material 1 [file mgen-6-455-s001.pdf]

Table S1: A list of the tetracycline resistance genes that were included in the three hidden Markov models.

Enzymatic genes		Ribosomal protection genes		Efflux genes	
Protein name	Accession no.	Protein name	Accession no.	Protein name	Accession no.
Tet(X)	AAA27471.1	Otr(A)	CAA37477.1	Tet(D)	CAA46706
Tet(X3)	BAC77725.1	Tet(Q)	CAA79727.1	Tet(B)	AAB59094
Tet(47)	AKQ05891.1	Tet(S)	AAA25293.1	Tet(H)	AAC43250
Tet(48)	AKQ05892.1	TetB(P)	AAA20117.1	Tet(Y)	AAC72341
Tet(49)	AKQ05893.1	Tet(M)	AAA73978.1	Tet(30)	AAD09860
Tet(50)	AKQ05894.1	Tet(T)	AAF01499.1	Tet(G)	AAC98496
Tet(51)	AKQ05895.1	Tet(O)	AAA23033.2	Tet(J)	AAD12753
Tet(52)	AKQ05896.1	Tet(36)	CAD55718.1	Tet(Z)	AAD25063
Tet(53)	AKQ05897.1	Tet(W)	CAA10975.1	Tet(31)	CAC80727
Tet(54)	AKQ05898.1	Tet(32)	ABG36114.2	Tet(33)	CAD12227
Tet(55)	AKQ05899.1	Tet(44)	CBH51823.1	Tet(A)	AAN06707
				Tet(39)	AAW66497
				Tet(41)	AAP93922
				Tet(42)	ACD35503
				Tet(57)	AJO67548
				Tet(59)	AMP42440
				Tet(E)	ABO92308
				Tet(C)	AAK97755

Table S2: Estimated sensitivity and specificity for the three created hidden Markov models.

Model	Reference genes	Sensitivity		Specificity	
		Full-length	Reads (100 nt)	Full-length	Reads (100 nt)
Enzyme	11	1	0.96	1	0.93
RPG	11	1	0.78	1	0.90
Efflux	18	0.94	0.89	1	0.97

Table S4: Distribution of identified previously known tetracycline resistance genes (>80% to any previously known tetracycline resistance gene) in the metagenomic datasets.

	Human Gut	HMP	Pig Gut	WWTP	Pune River	Polluted River
Ribosomal prot.						
Tet(33)			x	x		
Tet(39)				x	x	x
Tet(A)	x	x	x	x		x
Tet(B)	x	x	x		x	
Tet(G)			x		x	
Tet(H)			x			
Efflux						
Tet(32)	x	x	x		x	
Tet(36)	x				x	x
Tet(44)	x	x		x	x	
TetB(P)	x	x	x	x	x	
Tet(W)	x			x	x	
Tet(Q)	x				x	
Tet(T)			x		x	
Tet(S)			x	x		
Tet(O)	x				x	
Tet(M)	x					
Enzymatic						
Tet(X)	x	x		x	x	x

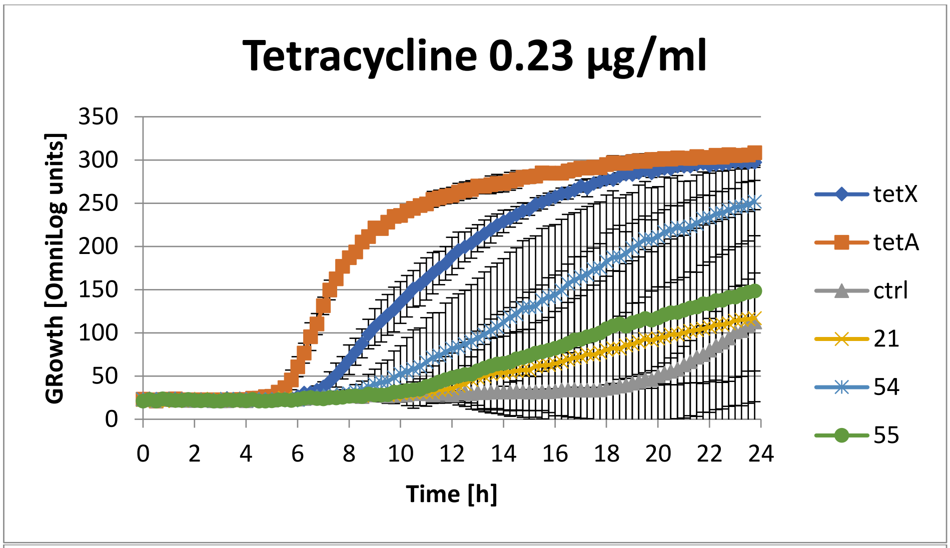

a)

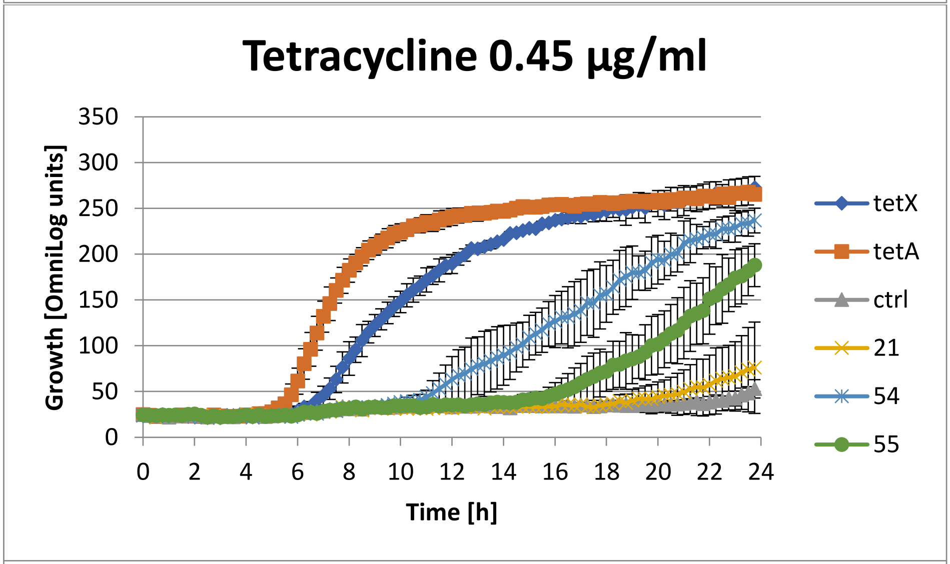

b)

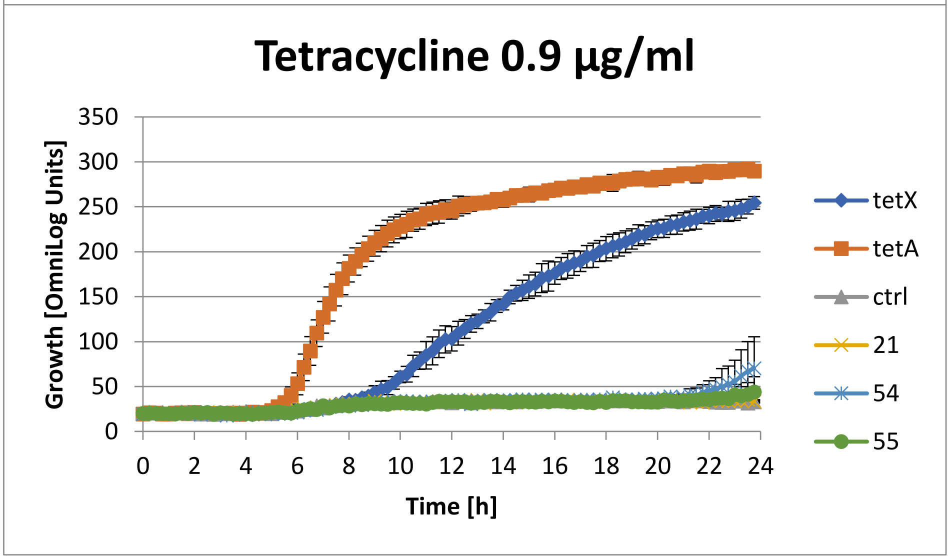

c)

Figure S1: Growth behavior of three constructs (efflux pump G241 in NF21, ribosomal protection G231 in NF54 and ribosomal protection G242 in NF55) compared to Tet(X), Tet(A) and a negative control at three different tetracycline concentrations.

Figure S2: Phylogenetic tree of the enzymatic degradation genes with bootstrap values.

Figure S3: Phylogenetic tree of the ribosomal protection genes with bootstrap values.

Figure S4: Phylogenetic tree of the efflux pump genes with bootstrap values.

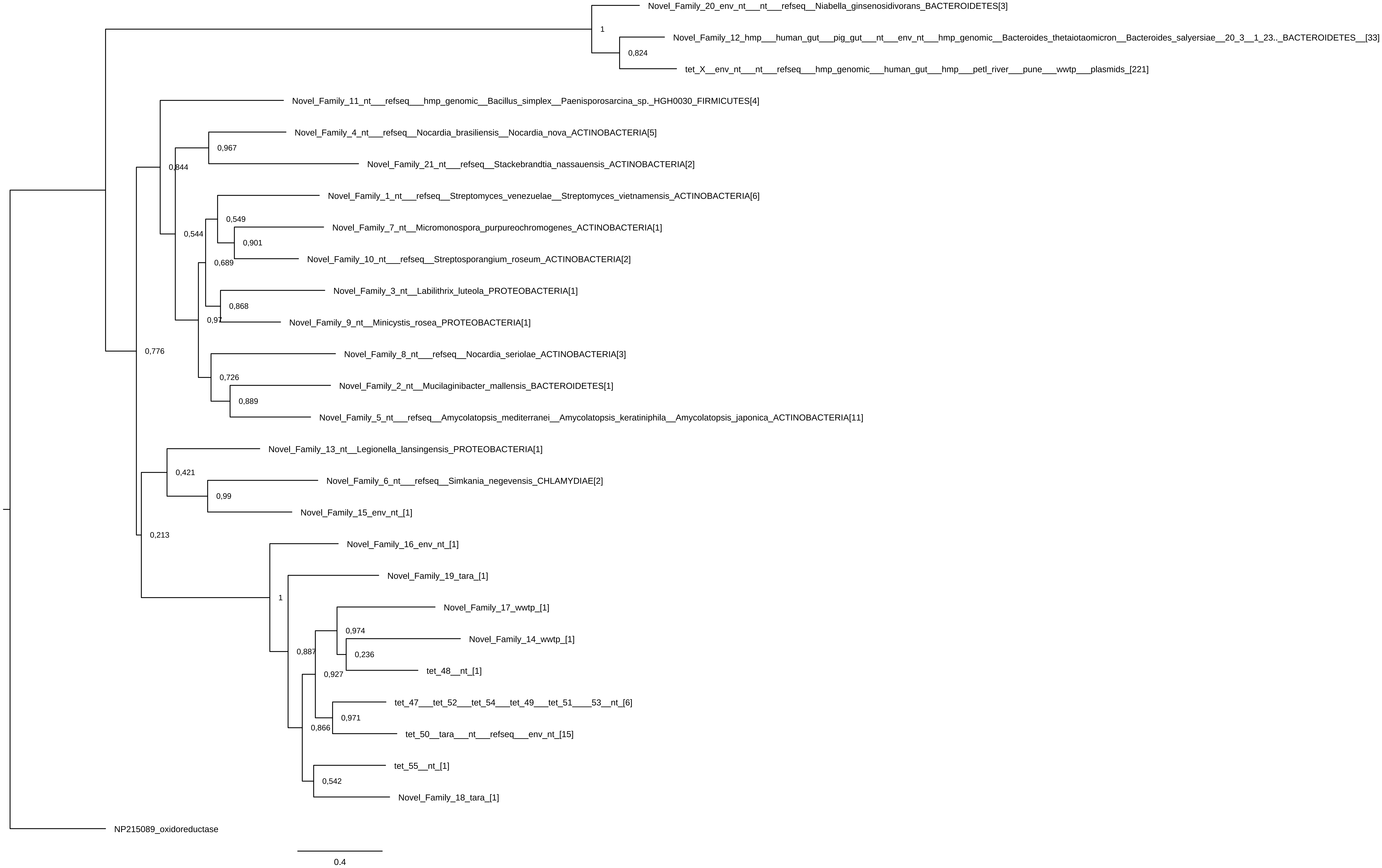
Novel_Family_20_env_nt___nt___refseq__Niabella_ginsenosidivorans_BACTEROIDETES[3]
1
Novel_Family_12_hmp___human_gut___pig_gut___nt___env_nt___hmp_genomic__Bacteroides_thetaiotaomicron__Bacteroides_salyersiae__20_3__1_23.._BACTEROIDETES__[33]
0,824
tet_X__env_nt___nt___refseq___hmp_genomic___human_gut___hmp___petl_river___pune___wwtp___plasmids_[221]
Novel_Family_11_nt___refseq___hmp_genomic__Bacillus_simplex__Paenisporosarcina_sp._HGH0030_FIRMICUTES[4]
Novel_Family_4_nt___refseq__Nocardia_brasiliensis__Nocardia_nova_ACTINOBACTERIA[5]
0,967
0,844
Novel_Family_21_nt___refseq__Stackebrandtia_nassauensis_ACTINOBACTERIA[2]
Novel_Family_1_nt___refseq__Streptomyces_venezuelae__Streptomyces_vietnamensis_ACTINOBACTERIA[6]
0,549
Novel_Family_7_nt__Micromonospora_purpureochromogenes_ACTINOBACTERIA[1]
0,544
0,901
Novel_Family_10_nt___refseq__Streptosporangium_roseum_ACTINOBACTERIA[2]
0,689
Novel_Family_3_nt__Labilithrix_luteola_PROTEOBACTERIA[1]
0,868
0,97
Novel_Family_9_nt__Minicystis_rosea_PROTEOBACTERIA[1]
0,776
Novel_Family_8_nt___refseq__Nocardia_seriolae_ACTINOBACTERIA[3]
0,726
Novel_Family_2_nt__Mucilaginibacter_mallensis_BACTEROIDETES[1]
0,889
Novel_Family_5_nt___refseq__Amycolatopsis_mediterranei__Amycolatopsis_keratiniphila__Amycolatopsis_japonica_ACTINOBACTERIA[11]
Novel_Family_13_nt__Legionella_lansingensis_PROTEOBACTERIA[1]
0,421
Novel_Family_6_nt___refseq__Simkania_negevensis_CHLAMYDIAE[2]
0,99
Novel_Family_15_env_nt_[1]
0,213
Novel_Family_16_env_nt_[1]
Novel_Family_19_tara_[1]
1
Novel_Family_17_wwtp_[1]
0,974
Novel_Family_14_wwtp_[1]
0,887
0,236
tet_48__nt_[1]
0,927
tet_47___tet_52___tet_54___tet_49___tet_51____53__nt_[6]
0,971
0,866
tet_50__tara___nt___refseq___env_nt_[15]
tet_55__nt_[1]
0,542
Novel_Family_18_tara_[1]
NP215089_oxidoreductase
0.4

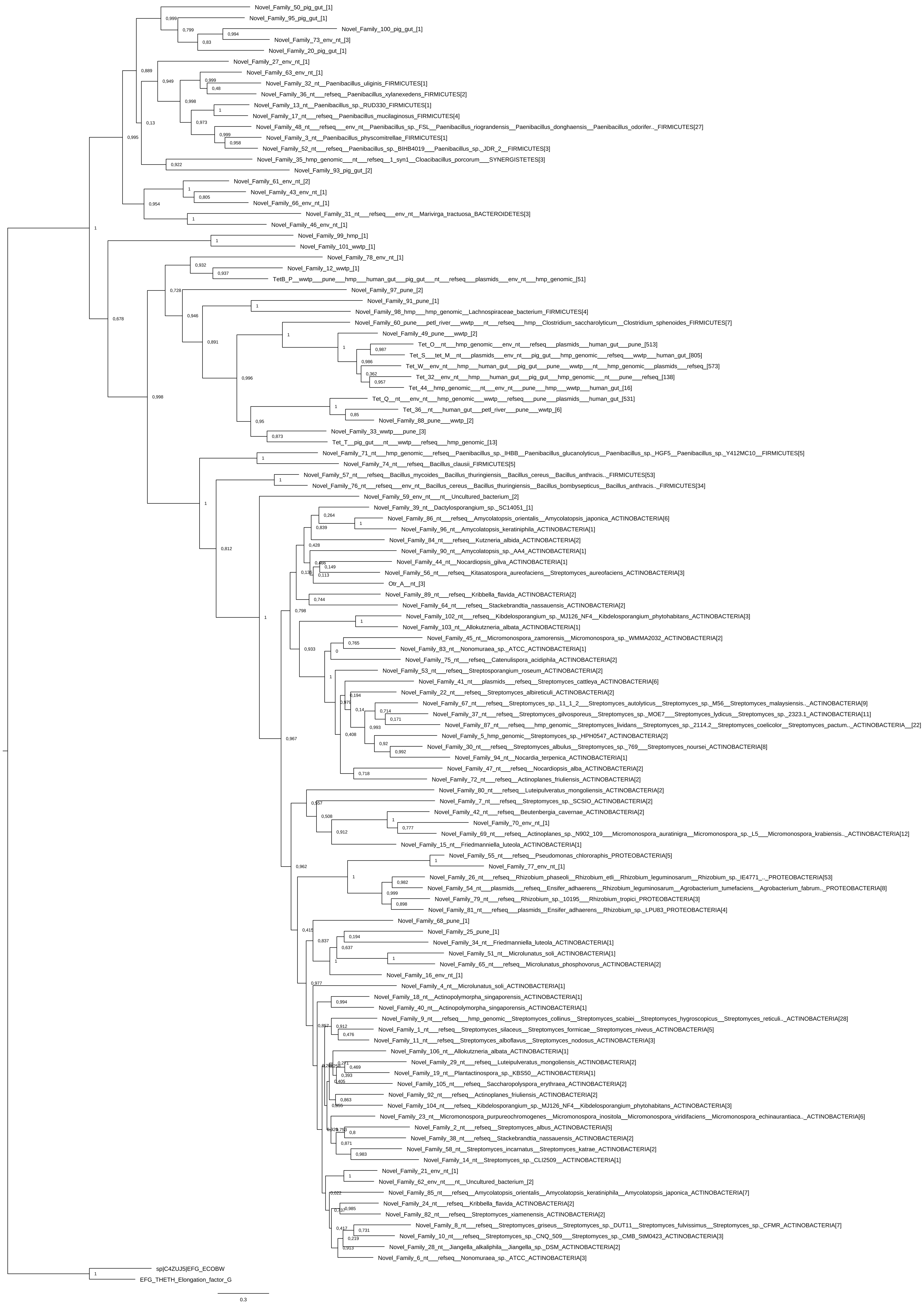
Novel_Family_50_pig_gut_[1]
Novel_Family_95_pig_gut_[1]
Novel_Family_100_pig_gut_[1]
Novel_Family_73_env_nt_[3]
Novel_Family_20_pig_gut_[1]
Novel_Family_27_env_nt_[1]
Novel_Family_63_env_nt_[1]
Novel_Family_32_nt__Paenibacillus_uliginis_FIRMICUTES[1]
Novel_Family_36_nt___refseq__Paenibacillus_xylanexedens_FIRMICUTES[2]
Novel_Family_13_nt__Paenibacillus_sp._RUD330_FIRMICUTES[1]
Novel_Family_17_nt___refseq__Paenibacillus_mucilaginosus_FIRMICUTES[4]
Novel_Family_48_nt___refseq___env_nt__Paenibacillus_sp._FSL__Paenibacillus_riograndensis__Paenibacillus_donghaensis__Paenibacillus_odorifer..._FIRMICUTES[27]
Novel_Family_3_nt__Paenibacillus_physcomitrellae_FIRMICUTES[1]
Novel_Family_52_nt___refseq__Paenibacillus_sp._BIHB4019___Paenibacillus_sp._JDR_2__FIRMICUTES[3]
Novel_Family_35_hmp_genomic___nt___refseq__1_syn1__Cloacibacillus_porcorum___SYNERGISTETES[3]
Novel_Family_93_pig_gut_[2]
Novel_Family_61_env_nt_[2]
Novel_Family_43_env_nt_[1]
Novel_Family_66_env_nt_[1]
Novel_Family_31_nt___refseq___env_nt__Marivirga_tractuosa_BACTEROIDETES[3]
Novel_Family_46_env_nt_[1]
Novel_Family_99_hmp_[1]
Novel_Family_101_wwtp_[1]
Novel_Family_78_env_nt_[1]
Novel_Family_12_wwtp_[1]
TetB_P__wwtp___pune___hmp___human_gut___pig_gut___nt___refseq___plasmids___env_nt___hmp_genomic_[51]
Novel_Family_97_pune_[2]
Novel_Family_91_pune_[1]
Novel_Family_98_hmp___hmp_genomic__Lachnospiraceae_bacterium_FIRMICUTES[4]
Novel_Family_60_pune___petl_river___wwtp___nt___refseq___hmp__Clostridium_saccharolyticum__Clostridium_sphenoides_FIRMICUTES[7]
Novel_Family_49_pune___wwtp_[2]
Tet_O__nt___hmp_genomic___env_nt___refseq___plasmids___human_gut___pune_[513]
Tet_S___tet_M__nt___plasmids___env_nt___pig_gut___hmp_genomic___refseq___wwtp___human_gut_[805]
Tet_W__env_nt___hmp___human_gut___pig_gut___pune___wwtp___nt___hmp_genomic___plasmids___refseq_[573]
Tet_32__env_nt___hmp___human_gut___pig_gut___hmp_genomic___nt___pune___refseq_[138]
Tet_44__hmp_genomic___nt___env_nt___pune___hmp___wwtp___human_gut_[16]
Tet_Q__nt___env_nt___hmp_genomic___wwtp___refseq___pune___plasmids___human_gut_[531]
Tet_36__nt___human_gut___petl_river___pune___wwtp_[6]
Novel_Family_88_pune___wwtp_[2]
Novel_Family_33_wwtp___pune_[3]
Tet_T__pig_gut___nt___wwtp___refseq___hmp_genomic_[13]
Novel_Family_71_nt___hmp_genomic___refseq__Paenibacillus_sp._IHBB__Paenibacillus_glucanolyticus__Paenibacillus_sp._HGF5__Paenibacillus_sp._Y412MC10__FIRMICUTES[5]
Novel_Family_74_nt___refseq__Bacillus_clausii_FIRMICUTES[5]
Novel_Family_57_nt___refseq__Bacillus_mycoides__Bacillus_thuringiensis__Bacillus_cereus__Bacillus_anthracis..._FIRMICUTES[53]
Novel_Family_76_nt___refseq___env_nt__Bacillus_cereus__Bacillus_thuringiensis__Bacillus_bombysepticus__Bacillus_anthracis..._FIRMICUTES[34]
Novel_Family_59_env_nt___nt__Uncultured_bacterium_[2]
Novel_Family_39_nt__Dactylosporangium_sp._SC14051_[1]
Novel_Family_86_nt___refseq__Amycolatopsis_orientalis__Amycolatopsis_japonica_ACTINOBACTERIA[6]
Novel_Family_96_nt__Amycolatopsis_keratiniphila_ACTINOBACTERIA[1]
Novel_Family_84_nt___refseq__Kutzneria_albida_ACTINOBACTERIA[2]
Novel_Family_90_nt__Amycolatopsis_sp._AA4_ACTINOBACTERIA[1]
Novel_Family_44_nt__Nocardiopsis_gilva_ACTINOBACTERIA[1]
Novel_Family_56_nt___refseq__Kitasatospora_aureofaciens__Streptomyces_aureofaciens_ACTINOBACTERIA[3]
Otr_A__nt_[3]
Novel_Family_89_nt___refseq__Kribbella_flavida_ACTINOBACTERIA[2]
Novel_Family_64_nt___refseq__Stackebrandtia_nassauensis_ACTINOBACTERIA[2]
Novel_Family_102_nt___refseq__Kibdelosporangium_sp._MJ126_NF4__Kibdelosporangium_phytohabitans_ACTINOBACTERIA[3]
Novel_Family_103_nt__Allokutzneria_albata_ACTINOBACTERIA[1]
Novel_Family_45_nt__Micromonospora_zamorensis__Micromonospora_sp._WMMA2032_ACTINOBACTERIA[2]
Novel_Family_83_nt__Nonomuraea_sp._ATCC_ACTINOBACTERIA[1]
Novel_Family_75_nt___refseq__Catenulispora_acidiphila_ACTINOBACTERIA[2]
Novel_Family_53_nt___refseq__Streptosporangium_roseum_ACTINOBACTERIA[2]
Novel_Family_41_nt___plasmids___refseq__Streptomyces_cattleya_ACTINOBACTERIA[6]
Novel_Family_22_nt___refseq__Streptomyces_albireticuli_ACTINOBACTERIA[2]
Novel_Family_67_nt___refseq__Streptomyces_sp._11_1_2___Streptomyces_autolyticus__Streptomyces_sp._M56__Streptomyces_malaysiensis..._ACTINOBACTERIA[9]
Novel_Family_37_nt___refseq__Streptomyces_gilvosporeus__Streptomyces_sp._MOE7___Streptomyces_lydicus__Streptomyces_sp._2323.1_ACTINOBACTERIA[11]
Novel_Family_87_nt___refseq___hmp_genomic__Streptomyces_lividans__Streptomyces_sp._2114.2__Streptomyces_coelicolor__Streptomyces_pactum..._ACTINOBACTERIA__[22]
Novel_Family_5_hmp_genomic__Streptomyces_sp._HPH0547_ACTINOBACTERIA[2]
Novel_Family_30_nt___refseq__Streptomyces_albulus__Streptomyces_sp._769___Streptomyces_noursei_ACTINOBACTERIA[8]
Novel_Family_94_nt__Nocardia_terpenica_ACTINOBACTERIA[1]
Novel_Family_47_nt___refseq__Nocardiopsis_alba_ACTINOBACTERIA[2]
Novel_Family_72_nt___refseq__Actinoplanes_friuliensis_ACTINOBACTERIA[2]
Novel_Family_80_nt___refseq__Luteipulveratus_mongoliensis_ACTINOBACTERIA[2]
Novel_Family_7_nt___refseq__Streptomyces_sp._SCSIO_ACTINOBACTERIA[2]
Novel_Family_42_nt___refseq__Beutenbergia_cavernae_ACTINOBACTERIA[2]
Novel_Family_70_env_nt_[1]
Novel_Family_69_nt___refseq__Actinoplanes_sp._N902_109___Micromonospora_auratinigra__Micromonospora_sp._L5___Micromonospora_krabiensis..._ACTINOBACTERIA[12]
Novel_Family_15_nt__Friedmanniella_luteola_ACTINOBACTERIA[1]
Novel_Family_55_nt___refseq__Pseudomonas_chlororaphis_PROTEOBACTERIA[5]
Novel_Family_77_env_nt_[1]
Novel_Family_26_nt___refseq__Rhizobium_phaseoli__Rhizobium_etli__Rhizobium_leguminosarum__Rhizobium_sp._IE4771_..._PROTEOBACTERIA[53]
Novel_Family_54_nt___plasmids___refseq__Ensifer_adhaerens__Rhizobium_leguminosarum__Agrobacterium_tumefaciens__Agrobacterium_fabrum..._PROTEOBACTERIA[8]
Novel_Family_79_nt___refseq__Rhizobium_sp._10195___Rhizobium_tropici_PROTEOBACTERIA[3]
Novel_Family_81_nt___refseq___plasmids___Ensifer_adhaerens__Rhizobium_sp._LPU83_PROTEOBACTERIA[4]
Novel_Family_68_pune_[1]
Novel_Family_25_pune_[1]
Novel_Family_34_nt__Friedmanniella_luteola_ACTINOBACTERIA[1]
Novel_Family_51_nt__Microlunatus_soli_ACTINOBACTERIA[1]
Novel_Family_65_nt___refseq__Microlunatus_phosphovorus_ACTINOBACTERIA[2]
Novel_Family_16_env_nt_[1]
Novel_Family_4_nt__Microlunatus_soli_ACTINOBACTERIA[1]
Novel_Family_18_nt__Actinopolymorpha_singaporensis_ACTINOBACTERIA[1]
Novel_Family_40_nt__Actinopolymorpha_singaporensis_ACTINOBACTERIA[1]
Novel_Family_9_nt___refseq___hmp_genomic__Streptomyces_collinus__Streptomyces_scabiei__Streptomyces_hygroscopicus__Streptomyces_reticuli..._ACTINOBACTERIA[28]
Novel_Family_1_nt___refseq__Streptomyces_silaceus__Streptomyces_formicae__Streptomyces_niveus_ACTINOBACTERIA[5]
Novel_Family_11_nt___refseq__Streptomyces_alboflavus__Streptomyces_nodosus_ACTINOBACTERIA[3]
Novel_Family_106_nt__Allokutzneria_albata_ACTINOBACTERIA[1]
Novel_Family_29_nt___refseq__Luteipulveratus_mongoliensis_ACTINOBACTERIA[2]
Novel_Family_19_nt__Plantactinospora_sp._KBS50__ACTINOBACTERIA[1]
Novel_Family_105_nt___refseq__Saccharopolyspora_erythraea_ACTINOBACTERIA[2]
Novel_Family_92_nt___refseq__Actinoplanes_friuliensis_ACTINOBACTERIA[2]
Novel_Family_104_nt___refseq__Kibdelosporangium_sp._MJ126_NF4__Kibdelosporangium_phytohabitans_ACTINOBACTERIA[3]
Novel_Family_23_nt__Micromonospora_purpureochromogenes__Micromonospora_inositola__Micromonospora_viridifaciens__Micromonospora_echinaurantiaca..._ACTINOBACTERIA[6]
Novel_Family_2_nt___refseq__Streptomyces_albus_ACTINOBACTERIA[5]
Novel_Family_38_nt___refseq__Stackebrandtia_nassauensis_ACTINOBACTERIA[2]
Novel_Family_58_nt__Streptomyces_incarnatus__Streptomyces_katrae_ACTINOBACTERIA[2]
Novel_Family_14_nt__Streptomyces_sp._CLI2509__ACTINOBACTERIA[1]
Novel_Family_21_env_nt_[1]
Novel_Family_62_env_nt___nt__Uncultured_bacterium_[2]
Novel_Family_85_nt___refseq__Amycolatopsis_orientalis__Amycolatopsis_keratiniphila__Amycolatopsis_japonica_ACTINOBACTERIA[7]
Novel_Family_24_nt___refseq__Kribbella_flavida_ACTINOBACTERIA[2]
Novel_Family_82_nt___refseq__Streptomyces_xiamenensis_ACTINOBACTERIA[2]
Novel_Family_8_nt___refseq__Streptomyces_griseus__Streptomyces_sp._DUT11__Streptomyces_fulvissimus__Streptomyces_sp._CFMR_ACTINOBACTERIA[7]
Novel_Family_10_nt___refseq__Streptomyces_sp._CNQ_509___Streptomyces_sp._CMB_StM0423_ACTINOBACTERIA[3]
Novel_Family_28_nt__Jiangella_alkaliphila__Jiangella_sp._DSM_ACTINOBACTERIA[2]
Novel_Family_6_nt___refseq__Nonomuraea_sp._ATCC_ACTINOBACTERIA[3]
sp|C4ZUJ5|EFG_ECOBW
EFG_THETH_Elongation_factor_G
0.3

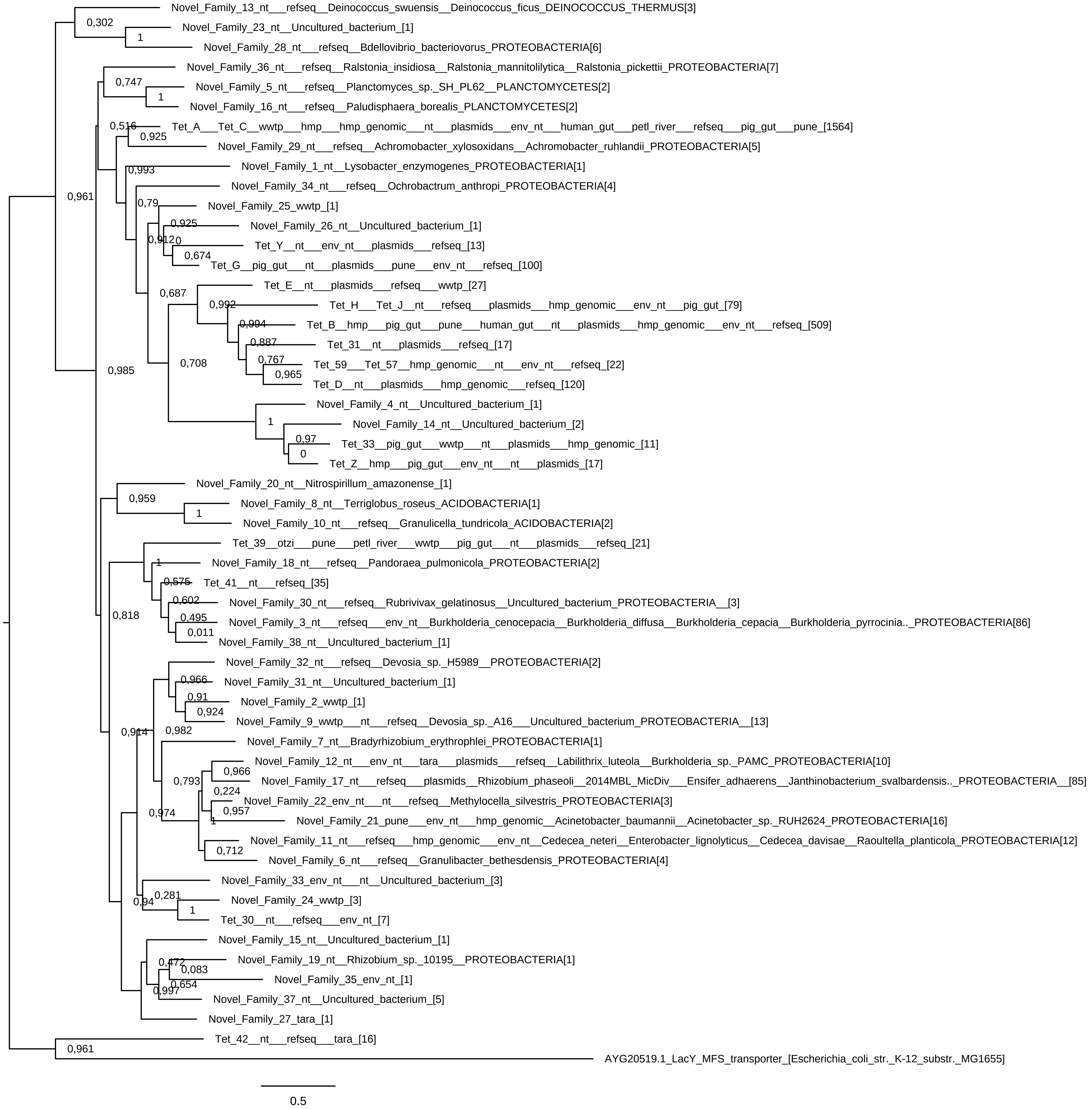

Novel_Family_13_nt___refseq__Deinococcus_swuensis__Deinococcus_ficus_DEINOCOCCUS_THERMUS[3]
0,302
Novel_Family_23_nt__Uncultured_bacterium_[1]
1
Novel_Family_28_nt___refseq__Bdellovibrio_bacteriovorus_PROTEOBACTERIA[6]
Novel_Family_36_nt___refseq__Ralstonia_insidiosa__Ralstonia_mannitolilytica__Ralstonia_pickettii_PROTEOBACTERIA[7]
0,747
Novel_Family_5_nt___refseq__Planctomyces_sp._SH_PL62__PLANCTOMYCETES[2]
1
Novel_Family_16_nt___refseq__Paludisphaera_borealis_PLANCTOMYCETES[2]
0,516
Tet_A___Tet_C__wwtp___hmp___hmp_genomic___nt___plasmids___env_nt___human_gut___petl_river___refseq___pig_gut___pune_[1564]
0,925
Novel_Family_29_nt___refseq__Achromobacter_xylosoxidans__Achromobacter_ruhlandii_PROTEOBACTERIA[5]
0,993
Novel_Family_1_nt__Lysobacter_enzymogenes_PROTEOBACTERIA[1]
0,961
Novel_Family_34_nt___refseq__Ochrobactrum_anthropi_PROTEOBACTERIA[4]
0,79
Novel_Family_25_wwtp_[1]
0,925
Novel_Family_26_nt__Uncultured_bacterium_[1]
0,912
0
Tet_Y__nt___env_nt___plasmids___refseq_[13]
0,674
Tet_G__pig_gut___nt___plasmids___pune___env_nt___refseq_[100]
Tet_E__nt___plasmids___refseq___wwtp_[27]
0,687
0,992
Tet_H___Tet_J__nt___refseq___plasmids___hmp_genomic___env_nt___pig_gut_[79]
0,994
Tet_B__hmp___pig_gut___pune___human_gut___nt___plasmids___hmp_genomic___env_nt___refseq_[509]
0,887
Tet_31__nt___plasmids___refseq_[17]
0,708
0,767
Tet_59___Tet_57__hmp_genomic___nt___env_nt___refseq_[22]
0,985
0,965
Tet_D__nt___plasmids___hmp_genomic___refseq_[120]
Novel_Family_4_nt__Uncultured_bacterium_[1]
1
Novel_Family_14_nt__Uncultured_bacterium_[2]
0,97
Tet_33__pig_gut___wwtp___nt___plasmids___hmp_genomic_[11]
0
Tet_Z__hmp___pig_gut___env_nt___nt___plasmids_[17]
Novel_Family_20_nt__Nitrospirillum_amazonense_[1]
0,959
Novel_Family_8_nt__Terriglobus_roseus_ACIDOBACTERIA[1]
1
Novel_Family_10_nt___refseq__Granulicella_tundricola_ACIDOBACTERIA[2]
Tet_39__otzi___pune___petl_river___wwtp___pig_gut___nt___plasmids___refseq_[21]
1
Novel_Family_18_nt___refseq__Pandoraea_pulmonicola_PROTEOBACTERIA[2]
0,575
Tet_41__nt___refseq_[35]
0,602
Novel_Family_30_nt___refseq__Rubrivivax_gelatinosus__Uncultured_bacterium_PROTEOBACTERIA__[3]
0,818
0,495
Novel_Family_3_nt___refseq___env_nt__Burkholderia_cenocepacia__Burkholderia_diffusa__Burkholderia_cepacia__Burkholderia_pyrrocinia.._PROTEOBACTERIA[86]
0,011
Novel_Family_38_nt__Uncultured_bacterium_[1]
Novel_Family_32_nt___refseq__Devosia_sp._H5989__PROTEOBACTERIA[2]
0,966
Novel_Family_31_nt__Uncultured_bacterium_[1]
0,91
Novel_Family_2_wwtp_[1]
0,924
Novel_Family_9_wwtp___nt___refseq__Devosia_sp._A16___Uncultured_bacterium_PROTEOBACTERIA__[13]
0,914
0,982
Novel_Family_7_nt__Bradyrhizobium_erythrophlei_PROTEOBACTERIA[1]
Novel_Family_12_nt___env_nt___tara___plasmids___refseq__Labilithrix_luteola__Burkholderia_sp._PAMC_PROTEOBACTERIA[10]
0,966
0,793
Novel_Family_17_nt___refseq___plasmids__Rhizobium_phaseoli__2014MBL_MicDiv___Ensifer_adhaerens__Janthinobacterium_svalbardensis.._PROTEOBACTERIA__[85]
0,224
Novel_Family_22_env_nt___nt___refseq__Methylocella_silvestris_PROTEOBACTERIA[3]
0,974
0,957
1
Novel_Family_21_pune___env_nt___hmp_genomic__Acinetobacter_baumannii__Acinetobacter_sp._RUH2624_PROTEOBACTERIA[16]
Novel_Family_11_nt___refseq___hmp_genomic___env_nt__Cedecea_neteri__Enterobacter_lignolyticus__Cedecea_davisae__Raoultella_planticola_PROTEOBACTERIA[12]
0,712
Novel_Family_6_nt___refseq__Granulibacter_bethesdensis_PROTEOBACTERIA[4]
Novel_Family_33_env_nt___nt__Uncultured_bacterium_[3]
0,281
0,94
Novel_Family_24_wwtp_[3]
1
Tet_30__nt___refseq___env_nt_[7]
Novel_Family_15_nt__Uncultured_bacterium_[1]
0,472
Novel_Family_19_nt__Rhizobium_sp._10195__PROTEOBACTERIA[1]
0,083
Novel_Family_35_env_nt_[1]
0,654
0,997
Novel_Family_37_nt__Uncultured_bacterium_[5]
Novel_Family_27_tara_[1]
Tet_42__nt___refseq___tara_[16]
0,961
AYG20519.1_LacY_MFS_transporter_[Escherichia_coli_str._K-12_substr._MG1655]
0.5
